# Obituary

**Published:** 1906-02-15

**Authors:** 


### OBITUARY.

D. H. Crouse, editor of the *Dental Digest*, died at his home in Chicago, January 3rd, after a short illness. This will be a great surprise and shock to the many friends of this active worker in the cause of dentistry, as he was a comparatively young man with promise of a long life of usefulness. Mr. Crouse was not a dentist, but he has been so intimately associated with his father Dr. J. N. Crouse in the great work that has consumed his time and thought for the last fifteen years, that he became thoroughly conversant with all public professional interests, and did much to help his father in organizing and carrying on the work of the Dental Protective Association. When the *Dental Digest* was issued, many were surprised that Dr. J. N. Crouse did not assume the editorship and management of the journal, but Howard, because of his literary training and love for that kind of work, has carried the burden of this work along with the duties of business manager of the Dental Supply Company. He was a genial and bright man socially and had many friends. The editor of the Register had very pleasant business and social relations with him, and it is with deep regret that we learn of his death. It is reportted that his heath was the result of over work and too close application to his business. His physicians. had warned him of an impending breakdown and he had made preparations for a period of rest and recuperation, but the warning came too late and he was called to rest, when it would seem that his work was unfinished. His great energy and determination have accomplished a task in a few years that many men would spend a life time at, with no greater results. He was a great comfort and helper to his father in the work which he has done for the love of his profession, and which has been so slightly appreciated by the mass of the profession. Every one who knows how close were the relations of the father and son in their business and social life will appreciate the deep sorrow that has come to one of the profession's greatest benefactors, and will unselfishly extend him in this great affliction most cordial sympathy.

